# MiR-509-3p is oncogenic, targets the tumor suppressor PHLPP2, and functions as a novel tumor adjacent normal tissue based prognostic biomarker in colorectal cancer

**DOI:** 10.1186/s12885-021-09075-x

**Published:** 2022-03-31

**Authors:** Deepak Narayanan Iyer, Dominic Chi-Chung Foo, Oswens Siu-Hung Lo, Timothy Ming-Hun Wan, Xue Li, Ryan Wai-Yan Sin, Roberta Wen-Chi Pang, Wai-Lun Law, Lui Ng

**Affiliations:** Department of Surgery, Li Ka Shing Faculty of Medicine, The University of Hong Kong, Hong Kong, China

**Keywords:** Colorectal cancer, microRNA, Prognosis, Tumor microenvironment

## Abstract

**Background:**

Recently the role of microRNAs has been explored immensely as novel regulators and potential biomarkers in several cancers. MiR-509-3p is one such miRNA that has been observed to show a mixed expression in different cancers, while it’s expression and clinical relevance in colorectal cancer (CRC) has not yet been characterized.

**Methods:**

We used quantitative PCR to evaluate the expression of miR-509-3p in fresh-frozen CRC tumor tissues and the corresponding tumor-adjacent normal (NAT) tissues from 103 patients. Subsequently, functional studies were performed to further interpret the role of the miRNA in CRC.

**Results:**

MiR-509-3p was found to be overexpressed in CRC tissues in nearly 80% of cases and was associated with an aggressive disease presentation. Notably, a higher expression of the miRNA promoted cell proliferation, migration, and invasion of CRC cells in in vitro and in vivo models. Mechanistically, we confirmed that miR-509-3p directly binds the 3’UTR of the tumor suppressor PHLPP2 and inhibits its expression. Furthermore, within the previous 103 clinical tissue specimens, we observed an overexpression of miR-509-3p within the NAT tissue of patients associated with a poor disease prognosis. Using multivariate analysis, it was observed that the expression of miR-509-3p within the NAT tissue was an independent predictor of prognosis in CRC. At the cellular level, through indirect coculture experiments, miR-509-3p was observed to regulate the proliferative, migratory, and invasive behavior of normal colon cells.

**Conclusion:**

MiR-509-3p strongly contributes to the development and progression of CRC and can potentially function as a prognostic biomarker in the disease.

**Supplementary Information:**

The online version contains supplementary material available at 10.1186/s12885-021-09075-x.

## Background

Notwithstanding the significant advancement in the diagnosis, classification, surgical and therapeutic strategies, effective clinical outcome of colorectal cancer (CRC) remains elusive [1]. Poor disease prognosis can be commonly attributed to disease recurrences that occurs in nearly 50% of the cases which lowers the median survival to less than 2 years, especially in patients with an advanced stage disease [2]. Not surprisingly, global numbers show that CRC is the third most prevalent cancer but ranks second in terms of cancer related deaths [3]. A better understanding of the molecular etiology and pathophysiology of CRC will further our understanding of critical markers of cancer development and progression which may lay foundation to improvement in disease monitoring and treatment approaches. Owing to years of research, we now know that CRC arises through a multistep process of sequential accumulation of genetic and epigenetic alterations that affect critical steps of tumor development including cell proliferation, survival, and metastasis. While mutations and aberrations in the function of several oncogenes and tumor suppressor genes associated with colorectal tumorigenesis have been identified and characterized, a significant array of other genetic and epigenetic factors responsible for disease progression remain largely unknown.

In the last 2–3 decades, the role of microRNAs (miRNAs) as a member of small endogenous non-coding RNAs that function as potent epigenetic regulators in diverse biological processes, has been explored immensely. Regulatory roles of miRNAs are commonly exerted by means of messenger RNA (mRNA) degradation or translational repression through the binding of specific sequence(s) within the 3′-untranslated regions (3′-UTR) of target mRNAs [4]. Target selection and consequently the expression levels, activity, and the functional significance is highly specific to each miRNA, and is dependent on the organ, tissue, or cell type under consideration [5, 6]. Interestingly, a wide majority of the human miRNAs are located within the cancer-related regions of the genome and function as critical regulators of oncogenes and tumor suppressor genes in several cancers [7–9]. Within CRC, thanks to deep profiling efforts numerous miRNAs have been identified, aberrant expression of which regulate cell proliferation, apoptosis, metastasis and other oncogenic pathways of cancer development and progression. Additionally, due to their small size (21–23 nucleotide), exceptional stability in multiple tissues and body fluids, and ease of detection and characterization through standard in vitro and in vivo assays, majority of these dysregulated miRNAs have also been shown to serve as valuable diagnostic and/or prognostic biomarkers in CRC. Nevertheless, there is still a major chunk of information regarding the regulation and function of several miRNAs associated with CRC development and progression that remains to be understood. Extensive investigations are consequently required to detect and characterize such miRNAs to further our understanding of the pathogenesis of CRC.

MiR-509-3p is one such small non-coding RNA that has not yet been characterized in CRC. Previous studies attempting to investigate the role of this miRNA in other cancers provide mixed reports of its behavior, ranging from a repressed expression in osteosarcoma, ovarian cancer, and glioma [10–12], to an overexpression in leukemia [13]. Nevertheless, there are numerous miRNAs reported in the literature that have an antagonistic behavior in different cancer types depending on the context [5, 6]. Svoronos et al cite several examples including miR-125b (oncogenic in hematological cancers and tumor suppressive in solid malignancies), miR-155 (tumor suppressive in gastric and ovarian cancer, oncomir in several cancers), and miR30b/d (tumor suppressive commonly, yet oncogenic in melanoma) that show contrasting behaviors in different cancers [14]. This evidence strongly supports the existence of multiple facets of miRNA activity in diverse cancers depending on several regulatory factors. Considering this scenario and the lack of any evidence of the role of this miRNA in CRC, we aimed to characterize and assess the clinical relevance of miR-509-3p within CRC. Initial investigations revealed that the miRNA shows an overexpression within CRC and correlates with an aggressive disease presentation. At the molecular level, miR-509-3p was found to directly target the tumor suppressor PH domain leucine-rich-repeats protein phosphatase 2 (PHLPP2) and inhibited its expression. A deeper clinical analysis subsequently showed that tumor adjacent normal (NAT) tissue expression of miR-509-3p is predictive for a poor disease outcome and can serve as a valuable prognostic biomarker in CRC.

## Methods

### Tissue specimens, cells, and animals

Prior to the usage of the clinical specimens for research purposes, ethical approval was attained from the University of Hong Kong’s Institutional review board (Reference number: UW 19-059, Date of approval: 17-Jan-2019). Besides, prior written informed consent was obtained from all patients recruited within the study. A total of 103 patients with histologically confirmed CRC who underwent surgical resection at the Department of Surgery, Queen Mary Hospital, University of Hong Kong were recruited for the study. Colorectal cancer resection specimens and tumor adjacent normal (NAT) tissues were collected, freshly frozen in liquid nitrogen and stored at -80 °C until further use. Medical records of the patients were reviewed to obtain all clinicopathological information relevant to the study. Disease recurrence or progression was monitored by assessing the patient’s radiographic scans. Assessment of disease prognosis was performed for at least a period of 5 years following primary curative surgery.

The human CRC cell lines HCT116 and SW480, and the normal colon cell line CCD841CoN, were all obtained from American Type Cell Culture (ATCC) and maintained in high glucose Dulbecco’s modified Eagle’s medium (DMEM) (Gibco/Thermofisher Scientific) supplemented with 10% fetal bovine serum (FBS), 100 U/ml penicillin, and 100 μg/ml streptomycin, in a humidified incubator at 37 °C with 5% CO_2_.

For the animal experiments, 4- to 6-week-old male NOD.CB17-Prkdcscid/J (NOD SCID) mice (The Jackson Laboratory, Bar Harbor, Maine) were purchased from Laboratory Animal Unit (LAU), University of Hong Kong, and were maintained in pathogen-free conditions. Animal care and experimental protocols were performed in accordance with the guidelines approved by the Committee on the Use of Live Animals in Teaching and Research (CULATR) of the University of Hong Kong.

### RNA extraction, cDNA synthesis and quantitative real time polymerase chain reaction

Total RNA was extracted from the fresh frozen colorectal tumor and the NAT tissue specimens using the mirVana™ miRNA Isolation Kit (Ambion/Thermofisher Scientific) according to the manufacturer’s guidelines. Extraction of total RNA from the cell lines was performed using the TRIzol® reagent (Invitrogen/Thermofisher Scientific) as per the manufacturer’s guidelines. For the quantitative estimation of miR-509-3p, the amplification primers as well as the universal oligo dT primer (for cDNA synthesis) were designed as per Balcells et al guidelines [15]. cDNA synthesis and quantitative PCR was conducted as per our previously published protocol [16]. Primer sequences used in the study are summarized in Supplementary Table 5.

### Plasmids, virus production and cell transduction

To generate a miR-509-3p expression vector, a 694 bp genomic fragment including the miR-509-3p pre-mir with an upstream and downstream overhang was PCR amplified and cloned into pCDH-cmv-mcs-EF1-copGFP vector (System Biosciences). Primer sequences used are: (5′-3′) Forward - AGCGAATTCTTAATGCTTTGCAAGTAGCAATG, Reverse - CGCGGATCCAATGAATAATACTCTTAAGGCAGAAAG. (Cloning sites are underlined – *Eco*RI (Forward), *Bam*HI (Reverse).

For a stable expression of miR-509-3p, the producer cell line 293TN (System Biosciences) was transfected with the lentiviral vector plasmid and ViraPower™ Lentiviral Packaging Mix (Invitrogen/Thermofisher Scientific) in the presence of Lipofectamine 3000 (Invitrogen/Thermofisher Scientific). After 48 h, the lentivirus-containing supernatant was sterile filtered and used to infect the CRC cell lines with polybrene (Sigma/Merck). Efficiency of viral transduction was determined by the intensity of fluorescence (GFP) expressed by the infected cells. Stably transduced cells were subsequently subjected to single cell dilution assay to select for cells showing maximum fluorescence intensity (gain-of-function). For the inhibition of the expression of miR-509-3p, a commercial antagomir against the miRNA (Shanghai Genepharma) was transfected in the miR-509-3p overexpressing CRC cell lines with Lipofectamine 3000 (Invitrogen/Thermofisher Scientific) as per manufacturer’s guidelines.

### Cell proliferation assay

CRC cell lines under study were seeded in triplicate in a 96-well flat bottom plate at a density of 3000 cells/well in a humidified incubator at 37 °C, 5% CO_2_ on day 0. At predetermined time intervals, cell proliferation was assessed with the aid of Cell proliferation ELISA, BrdU (colorimetric) kit (Roche) as per the manufacturer’s guidelines. For miR-509-3p inhibitor related cell proliferation assay, miR-509-3p (pre-mir) stably transduced CRC cell lines were transfected with the inhibitor, and then harvested after 24 h and subsequently used for cell proliferation assay. For coculture related cell proliferation assay, CCD841CoN cells were seeded (3000 cells/well) with conditioned media in the 96-well flat bottom plate on day 0. Cell proliferation was subsequently assessed like the previous protocol. For each cell type, absorbance data from triplicate experiments was expressed as a percentage of proliferating cells relative to the absorbance reads at the 24-h time point.

### Clonogenic assay

Cell lines were seeded in triplicate in 6-well plates (1000 cells/well) and incubated for 2–4 weeks. For miR-509-3p inhibitor related clonogenic assay, miR-509-3p (pre-mir) stably transduced CRC cell lines were transfected with the inhibitor, and then harvested after 24 h and subsequently used for the assay. Colonies were washed, fixed in ice-cold methanol, and stained with 0.1% crystal violet in ethanol. The wells were subsequently washed with water, air dried and the stained colonies were enumerated.

### Wound healing assay

For the wound healing assay, CCD841CoN cells were seeded in a 24-well plate until it achieved a 90–95% confluence, following which scratch wounds were produced using a 200 μl pipette tip across the length of the well. The cells were subsequently cultured in the conditioned media from miR-509-3p (pre-mir) or empty vector transduced CRC cell lines. Scratch wounds were observed at multiple time points and the distance between the scratch boundaries was measured to estimate the rate of wound closure and consequently the efficiency of cell migration.

### Transwell migration and invasion assay

For the transwell migration assay a 24-well Costar Transwell® Assay (8.0 μm pore size insert) (Corning/Merck) was used, while the transwell invasion assay was performed using 24-well Biocoat™ Matrigel™ Invasion Chamber Assay (8.0 μm pore size insert) (BD Biosciences) as per the manufacturer’s guidelines. For either assay, CRC cell lines under study were seeded (100,000 cells/upper chamber) in duplicate on day 0, in DMEM with 1% FBS and 100 U/ml penicillin, and 100 μg/ml streptomycin. In contrast, the lower chamber was flooded with the same medium containing 10% FBS which served as the chemoattractant. For the miRNA inhibitor related migration/invasion assay, miR-509-3p (pre-mir) stably transduced CRC cell lines were transfected with the inhibitor, harvested after 24 h and subsequently used for the migration or invasion assay. For the coculture migration/invasion assay, CCD841CoN was seeded in the upper chamber (100,000 cells/insert) in conditioned DMEM media with 1% FBS and antibiotics, while the lower chamber contained the same media with 10% FBS as a chemoattractant. Following an incubation period of 48 h at 37 °C, 5% CO_2_, unmigrated or uninvaded cells within the upper chamber were scraped using a cotton swab. The translocated cells at the bottom of the upper chamber were fixed in ice-cold methanol, stained in 0.1% crystal violet, washed and imaged. Images were taken at six random fields and the number of translocated cells were counted manually and expressed as an average fold-change from three independent experiments relative to the negative control.

### Western blotting

Cells were lysed using 1X RIPA buffer (Cell Signaling Technology) mixed with 1X protease inhibitor cocktail and 1 mmol/l phenylmethylsulfonyl fluoride. Protein concentrations were determined using Pierce BCA protein assay (Thermofisher Scientific) as per kit instructions. Equal amounts of proteins were loaded, resolved using 10% sodium dodecyl sulfate-polyacrylamide gel electrophoresis, transferred to polyvinylidene difluoride membranes, and incubated with primary antibodies: anti-E-cadherin (Cell Signaling Technology), anti-RhoA (Cell Signaling Technology), anti-actin (Santa Cruz Biotechnology), then incubated with corresponding HRP-conjugated secondary antibodies: anti-rabbit IgG (for anti-E-cadherin and anti-RhoA) (Cell Signaling Technology), and anti-goat IgG (for anti-actin) (Santa Cruz Biotechnology), and detected by enhanced chemiluminescence using ECL Western Blotting System (Amersham), on an X-ray film (Fumingwei).

### Target prediction and luciferase assay

Potential gene targets of miR-509-3p were predicted using miRmap (mirmap.ezlab.org) [17], TargetScanHuman (www.targetscan.org) [18], miRWalk (mirwalk.umm.uni-heidelberg.de/) [19], RNA22 (cm.jefferson.edu/rna22/) [20] and mirDIP (ophid.utoronto.ca/mirDIP/) [21, 22]. Gene targets predicted by at least 3 databases were shortlisted for subsequent analysis.

Probable target binding sites of miR-509-3p within the 3′-UTR regions of PHLPP2 was determined by the TargetScanHuman database (www.targetscan.org) [18]. Later, a 25 bp oligo duplex comprising of the original or mutant 3′-UTR binding regions of PHLPP2 was cloned into a pMIR-REPORT Luciferase vector (Thermofisher Scientific). Cystic Fibrosis Transmembrane Regulator (CFTR), a previously validated miR-509-3p target, was used as a positive control [23, 24]. For the Luciferase assay, HCT116 cells were seeded in triplicate in 24-well plates (100,000 cells/well). Next day, wild type or mutant reporter vectors with miR-509-3p mimic or scrambled control were cotransfected into the cells with the pRL-TK Renilla Luciferase control plasmid (Promega) using Lipofectamine 3000 reagent (Invitrogen/Thermofisher Scientific). After 48 h, Luciferase and Renilla activities were assayed using Dual-Luciferase Reporter Assay System (Promega) in a CLARIOstar multi-mode microplate reader (BMG Labtech) as per the manufacturer’s guidelines. Data was expressed as an average ratio of Firefly Luciferase versus Renilla Luciferase signal (Relative Luciferase Units) from three independent experiments.

### Animal experiments

HCT116 cells stably transduced with miR-509-3p (pre-mir) or empty vector were transfected with a Firefly luciferase expressing plasmid and selected by puromycin. For the animal experiments, Luc-HCT116 cells (5,000,000) stably transduced with miR-509-3p (pre-mir) (*N* = 7) or empty vector (*N* = 4) were injected orthotopically into the cecal wall of the NOD/SCID mice. Sample size used per study arm were selected randomly to achieve a basic statistical power for evaluation. After ~ 4 weeks, the mice were euthanized by rendering them unconscious first using Sodium Pentobarbital (40–60 mg/kg IP) followed by cervical dislocation. They were subsequently dissected, and tumor development was observed by capturing the bioluminescence signal using the PE IVIS Spectrum in vivo imaging system (PerkinElmer).

### Statistical analysis

Statistical analyses were conducted using Prism 8.0.1 (GraphPad Software Inc.) or SigmaPlot 14 (Systat Software Inc.) or SPSS 24 (IBM). For each dataset, normal distribution was assessed using the Shapiro-Wilk test prior to analysis. Data between two groups were compared using two-tailed Student’s t-test (paired or unpaired). For analyzing the relationship of miR-509-3p with the clinicopathological features of CRC, Student’s t-test or Mann-Whitney U test was used for comparing two sample groups. One-way ANOVA or Kruskal–Wallis test was applied for three or more sample groups. Survival curves were plotted by the Kaplan-Meier method and assessed by the log-rank test. Multivariate analysis was performed using a Cox proportional hazards model. A *p* value < 0.05 was considered statistically significant.

## Results

### MiR-509-3p is frequently overexpressed in CRC tissues and its high levels correlate with an aggressive disease

Clinicopathological information regarding patients recruited for this study is presented in Supplementary Table 1. Majority of the patients were male (61.2%), with a median age of 64 years. Within the study cohort, the colon (68%) served as the frequent site of tumor presentation followed by rectum (22.3%) and rectosigmoid (9.7%). While most patients commonly presented with a stage III disease (52/103), rest of the cases were almost equally distributed between early stages – stage I and II combined (28/103) and stage IV (23/103).

**Fig. 1:**
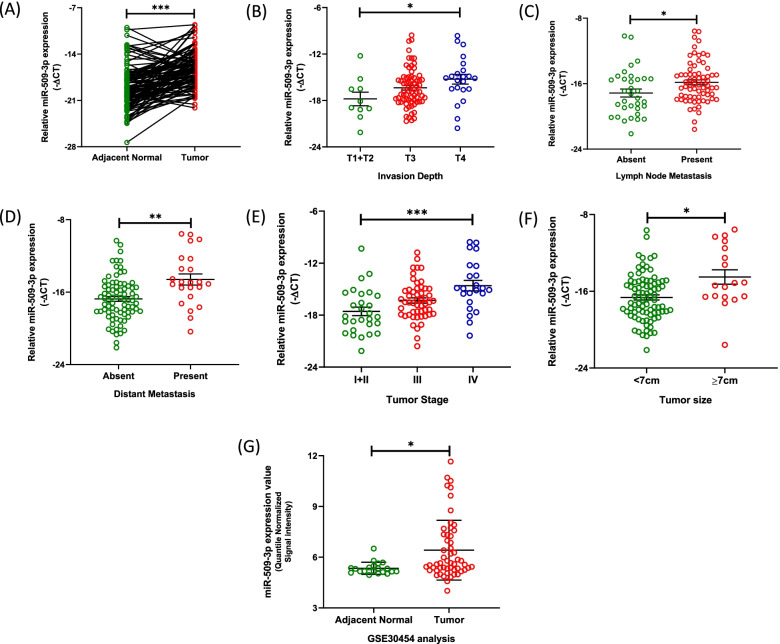
MiR-509-3p is overexpressed in CRC and correlates with an aggressive disease phenotype. **A** Real-time PCR-based expression analysis of miR-509-3p in CRC tumor and NAT tissues in 103 patients. **B**-**F** Correlation of the expression of miR-509-3p within the CRC tumor tissue with the clinicopathological characteristics of CRC patients within the study (*N* = 103) - **B** Tumor Invasion Depth, **C** Lymph Node Metastasis, **D** Distant Metastasis, **E** Tumor Stage, and **F** Tumor Size. For (**A**-**F**), expression of miR-509-3p was normalized to the expression of RNU6B. Expression results are shown as -∆CT. **F** Quantile normalized expression values of miR-509-3p in 20 colonic normal and 54 colorectal tumor samples obtained from the gene expression dataset (ID: GSE30454), publicly deposited within the NCBI’s Gene Expression Omnibus. *p value < 0.05; **p value < 0.01; ***p value < 0.001

To interpret the role of miR-509-3p in CRC, we used real time PCR to quantify the expression of the miRNA in the 103 colorectal tumor specimens and matched NAT tissues. The expression of miR-509-3p was normalized to the expression of RNU6B within the corresponding tissue type and expressed as -∆CT. Additionally, clinicopathological significance of the expression levels of miR-509-3p within the colorectal tumor tissue was also determined (Supplementary Table 2). MiR-509-3p was found to be frequently overexpressed in the colorectal tumor relative to the paired non-tumor mucosae in nearly 80% (82/103, fold change> 1.5) of cases (median: − 16.5 (tumor) vs. -19.33 (NAT); *p* < 0.0001) (Fig. 1A). Furthermore, a high expression of the miRNA positively correlated with a greater depth of tumor invasion (*p* = 0.0356, Fig. 1B) as well as the presence of nodal metastases (*p* = 0.0251, Fig. 1C) within the CRC patients. We also observed a remarkably higher expression of miR-509-3p in patients exhibiting distant metastases (18/23 cases with high miR-509-3p expression, 78.2%, median -∆CT: − 14.93), as compared to the nonmetastatic patient group (33/80 cases with high miR-509-3p expression, 41.2%, median -∆CT: − 16.85) (*p* = 0.0030) (Fig. 1D). Since a positive correlation was observed between an upregulated tumor expression of miR-509-3p and the presence of increasing tumor invasion, nodal, and distant metastases, consequently we also found a higher expression of the miRNA in CRC patients presenting with an advanced AJCC (8th edition) stage (*p* = 0.0003) (Fig. 1E). Moreover, CRC patients presenting with large primary tumors (≥7 cm) also showed a higher expression of miR-509-3p (*p* = 0.0138) (Fig. 1F). None of the other clinicopathological factors, such as age, gender, tumor location, or differentiation, showed any significant correlation with an upregulated tumor expression of miR-509-3p (Supplementary Table 2).

To validate the overexpression of miR-509-3p in CRC, we profiled a publicly deposited gene expression dataset - GSE30454 - from the NCBI’s Gene Expression Omnibus [25]. This dataset comprised of genome-wide miRNA expression profiles from a miRNA microarray-based analysis of 20 normal colonic and 54 primary colorectal tumor tissues. By comparing the quantile normalized signal intensities of miR-509-3p, we observed that the miRNA had an upregulated expression by 2.13-fold within the colorectal tumor tissues as compared to the normal colon (*p* = 0.0155) (Fig. 1E). Taken together, miR-509-3p was observed to be frequently overexpressed within the colorectal tumor relative to the normal colon.

### High expression of miR-509-3p is associated with an increased propagation of CRC cells

To validate the functional significance of an increased expression of miR-509-3p in CRC, we cloned the pre-mir sequence (hsa-mir-509-1) of the miRNA within a GFP-tagged lentiviral expression vector that was subsequently transduced into CRC cell lines - HCT116 and SW480, to generate a stable overexpression of miR-509-3p. Contrarily, to downregulate the miRNA, we used a commercial antagomir that specifically inhibited the expression of miR-509-3p within the miR-509-3p stably overexpressing CRC cell lines.

**Fig. 2:**
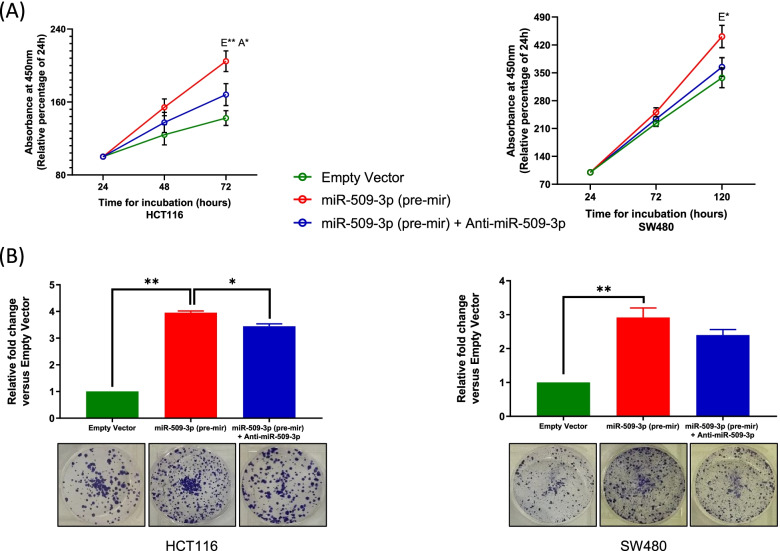
Upregulation of miR-509-3p induces cell proliferation and viability of CRC cells. Overexpression of miR-509-3p within stably transduced HCT116 and SW480 CRC cell lines resulted in an increase in their **A** proliferation rates – established using a BrdU assay, and **B** survival ability – established using a Clonogenic assay. E – relative to empty vector group, A – relative to (miR-509-3p (pre-mir) + Anti-miR-509-3p) group. *p value < 0.05; **p value < 0.01

Using a bromodeoxyuridine (BrdU) incorporation assay, we observed that an increased expression of miR-509-3p resulted in a higher rate of cell proliferation in the HCT116 (*p =* 0.0068) cell lines as compared to the negative control (Fig. 2A). This effect was subsequently reversed by the administration of an anti-miR-509-3p (*p* = 0.0106). Within SW480, while an increased rate of cell proliferation was observed upon miR-509-3p overexpression (*p* = 0.0272), the addition of an anti-miR-509-3p caused a reduction in the cell proliferation rates, although the miRNA-inhibition effect was not statistically significant (*p* = 0.0568).

To evaluate the effects of miR-509-3p on in vitro tumorigenesis, we measured the clonogenic survival ability of miR-509-3p through a colony formation assay. Approximately one thousand HCT116 or SW480 cells stably transduced with miR-509-3p, or empty vector control were cultured and maintained for a period of ~ 4 weeks. In the presence of elevated levels of miR-509-3p, the HCT116 cell line showed a 3.95-fold higher number of colonies as compared to the negative control (*p =* 0.0050). Moreover, in the presence of anti-miR-509-3p, there was a marked reduction in the number of HCT116 colonies as compared to the miR-509-3p overexpressing group (*p* = 0.0190) (Fig. 2B). For the SW480 cell line as well, we observed a significantly higher number of colonies formed upon overexpression of miR-509-3p (*p =* 0.0078). Although treatment with an anti-miR-509-3p caused a reduction in the number of colonies formed (2.39-fold (anti-miR-509-3p group) vs. 2.91-fold (miR-509-3p group) - as compared to the empty vector group), the effect was not statistically significant (*p =* 0.0553) (Fig. 2B).

These results indicated that miR-509-3p promotes cell proliferation as well as the survival ability of CRC cell lines, thereby ascertaining the importance of this miRNA in CRC tumorigenesis.

### MiR-509-3p increases the metastatic potential of CRC cell lines by targeting critical components of the epithelial-mesenchymal transition pathway

**Fig. 3:**
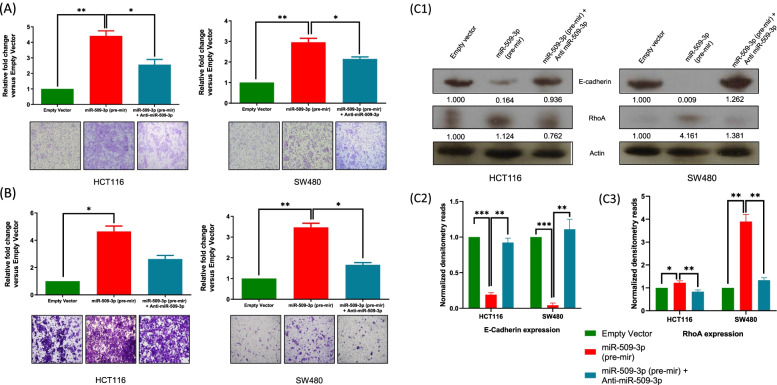
Overexpression of miR-509-3p induces CRC cell migration and invasion by regulating critical EMT pathway genes. MiR-509-3p stable transfectants showed increased **A** cell migration, and **B** cell invasion as compared with the vector control. The migration and invasion rates significantly decreased upon administration of an inhibitor against miR-509-3p. **C1–3** Western blotting analysis of indicated proteins. Expression of actin was used as a normalizer. Bar charts show the normalized densitometry values for E-Cadherin and RhoA expression within the study groups. *p value < 0.05; **p value < 0.01; ***p value < 0.01

Since miR-509-3p showed a positive clinical correlation with the presence of distant metastases, we wanted to characterize its potential in impacting the migration and invasion potential of CRC cell lines. Through a transwell migration assay, we observed that an upregulated expression of miR-509-3p, relative to the empty vector control, strongly increased the migratory potential of HCT116 (4.41-fold, *p* = 0.0089) and SW480 (2.96-fold, *p* = 0.0093) (Fig. 3A). Furthermore, targeted inhibition of miR-509-3p reduced the number of cells migrating across the transwell membrane for both HCT116 (*p* = 0.0128) and SW480 (*p* = 0.0361) cell lines, as compared to the miR-509-3p overexpressing group.

Likewise, we observed a higher proportion of miR-509-3p overexpressing HCT116 (*p* = 0.0118) and SW480 (*p* = 0.0065) cells invading through a transwell pre-coated with Matrigel™, as compared to the negative control (Fig. 3B). Knockdown of miR-509-3p caused a drop in the invasive potential of the CRC cell lines, specifically within the SW480 cells (*p* = 0.0276).

A critical step in cancer metastasis is Epithelial Mesenchymal Transition (EMT). The EMT mechanism involves an interplay of several factors that collaboratively cause the cancer cell to lose its epithelial morphology and form undifferentiated, mesenchymal-like cells that are associated with elevated migratory and invasive features [26]. To identify the molecular mechanisms associated with an increase in the metastatic potential of CRC cell lines under an elevated expression of miR-509-3p, we attempted to probe the expression of crucial genes involved in the EMT pathway. Through Western blotting, we observed that an overexpression of miR-509-3p within the CRC cell lines caused a significant decrease in the expression of epithelial cadherin (E-cadherin) with a concurrent increase in the expression of Ras Homolog Family Member A (RhoA) protein (Figure 3C1-C3), relative to the negative control. Parallelly, inhibition of miR-509-3p reversed the expression of E-Cadherin and RhoA proteins to the empty vector control defined protein expression baseline.

### PHLPP2 is a direct target of miR-509-3p

**Fig. 4:**
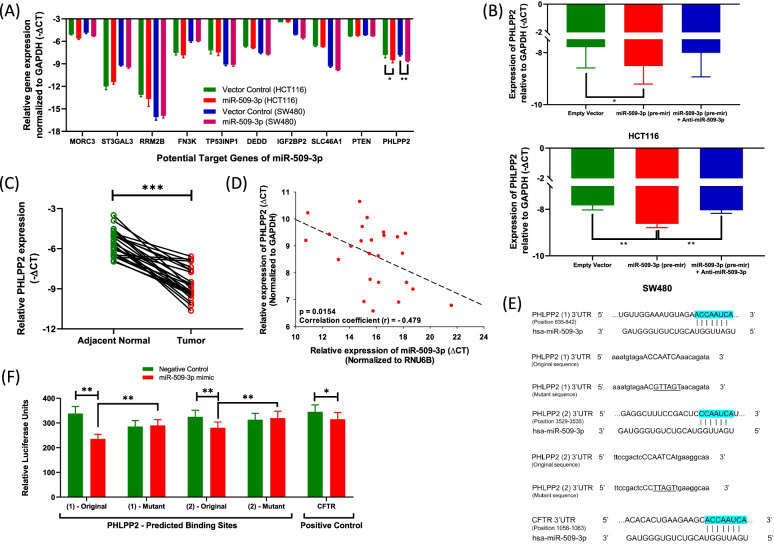
MiR-509-3p inhibits the expression of PHLPP2. **A** A list of top 10 predicted miR-509-3p target genes were probed in HCT116 or SW480 CRC cell lines stably transfected with miR-509-3p (pre-mir) or empty vector control using qPCR. Only one gene, PHLPP2 was found to be downregulated in both cell lines transfected with miR-509-3p as compared to negative control. **B** Upon administration of an inhibitor against miR-509-3p, the expression levels of PHLPP2 were restored. **C** Real-time PCR-based expression analysis of PHLPP2 in CRC tumor and NAT tissue in 25 patients. **D** Expression of PHLPP2 negatively correlated with the expression of miR-509-3p within the 25 CRC patients. For (**A**-**D**), expression of miR-509-3p was normalized to the expression of RNU6B, while expression of PHLPP2 and other putative target genes were normalized to the expression of GAPDH. Expression results are shown as -∆CT. **E** Two predicted binding sites for miR-509-3p within the 3′-UTR of PHLPP2 were identified by TargetScan. Within the original sequence, the uppercase characters indicate the original putative binding site of miR-509-3p within the 3′-UTR of PHLPP2. Within the mutant sequences, the underlined characters indicate the mutated bases within the potential binding site. Lowercase characters are the flanking 3′-UTR bases. CFTR was used as the positive control within the Luciferase Reporter assay. **F** HCT116 cells were co-transfected with PHLPP2 (1) or (2) 3′-UTR original or mutant sequence reporter constructs with miR-509-3p mimic or empty vector control and Renilla Luciferase control vector. For each transfectant, Firefly Lucferase signal was normalized with the Renilla Luciferase signal and expressed as Relative Luciferase Units. *p value < 0.05; **p value < 0.01; ***p value < 0.001

To further understand the molecular basis of the potentially oncogenic behavior of miR-509-3p within CRC, we attempted to identify putative gene targets of the miRNA by exploring multiple target prediction tools including miRmap, TargetScan, miRWalk, RNA22 and mirDIP. The top 10 potential targets projected by at least 3 different prediction algorithms were subsequently shortlisted based upon literature evidence of their involvement in the process of CRC carcinogenesis. These targets included - MORC Family CW-Type Zinc Finger 3 (MORC3), ST3 Beta-Galactoside Alpha-2,3-Sialyltransferase 3 (ST3GAL3), Ribonucleotide Reductase Regulatory TP53 Inducible Subunit M2B (RRM2B), Fructosamine 3 Kinase (FN3K), Tumor Protein P53 Inducible Nuclear Protein 1 (TP53INP1), Death Effector Domain Containing Protein (DEDD), Insulin Like Growth Factor 2 MRNA Binding Protein 2 (IGF2BP2), Solute Carrier Family 46 Member 1 (SLC46A1), Phophatase and Tensin Homolog (PTEN) and, PH domain leucine-rich repeat-containing protein phosphatase 2 (PHLPP2). Using real time PCR, we checked the expression of the putative targets in CRC cell lines stably transduced with miR-509-3p (pre-mir) or vector control. The expression of majority of the predicted targets was not influenced by an increased expression of miR-509-3p (Fig. 4A). However, only one target – PHLPP2 – showed a significant decrease in both HCT116 (*p* = 0.0122) and SW480 (*p* = 0.0058) cell lines stably overexpressing miR-509-3p (Fig. 4A & B). Contemporaneously, targeted inhibition of the miRNA by an antagomir caused an increase in the expression levels of PHLPP2 specifically within the SW480 cell line *(p* = 0.0010). Furthermore, in contrast to miR-509-3p, PHLPP2 was found to be significantly downregulated within the colorectal tumor tissues as compared to the adjacent normal (median: − 8.984 (tumor) vs. -5.611 (NAT); *N* = 25; *p* < 0.0001) (Fig. 4C). Additionally, the decreased expression of PHLPP2 within the tumor tissues was found to bear a negative correlation with the expression of miR-509-3p within the same clinical samples (*r* = − 0.479, *p* = 0.0154) (Fig. 4D).

To investigate the existence of a direct interaction between miR-509-3p and PHLPP2, we performed a dual luciferase reporter assay. The PHLPP2 3′-UTR containing the potential miR-509-3p binding sites and their corresponding mutant version (Fig. 4E) were cloned into a pMIR-REPORT vector and transfected into HCT116 cells with miR-509-3p mimic or empty vector. Cystic Fibrosis Transmembrane Regulator (CFTR) was used a positive control, since it was previously validated as a direct target of miR-509-3p [23, 24]. As a principle, binding of the 3′-UTR to the target region caused a repression in the luciferase expression. The results demonstrated a repression of luciferase expression for the positive control as well as the PHLPP2 3′-UTR binding sites in the presence of the miRNA mimic (Fig. 4F) relative to the negative control. Mutation of the putative binding sites relieved the repression by miR-509-3p, suggesting that the repression of messenger RNA (mRNA) expression was site-specific. These findings demonstrate that PHLPP2 is a direct functional target of miR-509-3p, inhibition of which may strongly contribute towards colorectal carcinogenesis.

### Upregulated expression of miR-509-3p results in an aggressive colorectal tumorigenesis in vivo

**Fig. 5:**
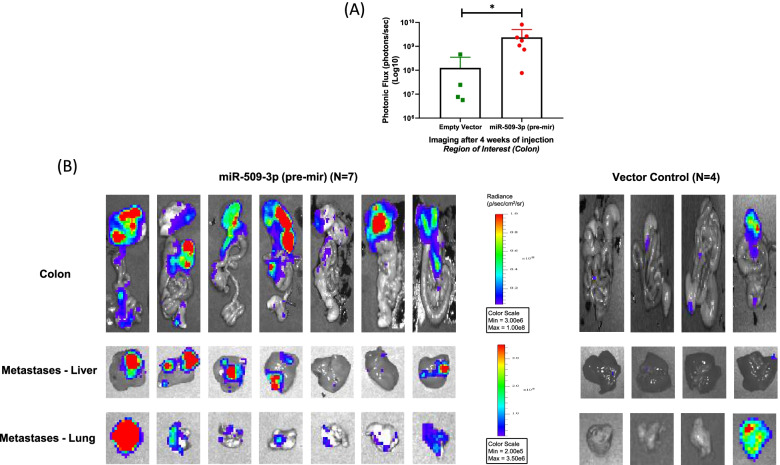
MiR-509-3p overexpressing mice develop larger tumors that have a higher propensity to form distant metastases. **A** Colon from mice orthotopically injected with stable HCT116-miR-509-3p (pre-mir) (*N* = 7) or HCT116-Empty Vector Control (*N* = 4) were assessed after 4 weeks of injection. Overexpression of miR-509-3p resulted in larger tumors showing a greater luciferase flux intensity as compared to the vector control. *p value < 0.05 **B** Strong to moderate luciferase signals were identified in distant organs (lungs and liver) for all the miR-509-3p (pre-mir) injected mice (*N* = 7). For the mice injected with vector control, majority of the distant sites (liver and lungs) showed very weak or no luciferase signals

To explore the role of miR-509-3p in the development and progression of CRC in vivo, we orthotopically injected 1 million luciferase-labelled HCT116 cells stably transduced with miR-509-3p (pre-mir) or empty vector control into the cecal wall of NOD-SCID mice. After ~ 4 weeks, mice from both groups developed primary tumors in the injected areas. Although, the miR-509-3p overexpressing mice group (*N* = 7) showed a higher luciferase signal within the colonic region relative to the control group (*N* = 4) mice (*p* = 0.0121), indicative of larger/aggressive primary tumors (Fig. 5A & B). Furthermore, within the seven mice injected with miR-509-3p-HCT116 cell line, luciferase signal was observed in livers (five mice with strong signal and two mice with weak signal) and lungs (strong signal in all mice) of all mice, suggesting that the primary tumor had metastasized to these sites (Fig. 5B). In contrast, the control mice showed weak liver metastasis in three out of four mice and lung metastasis in only one out of four mice (Fig. 5B). Put together, our results provided critical evidence that overexpression of miR-509-3p causes the development of aggressive CRC with distant metastasis in vivo.

### MiR-509-3p is overexpressed within the NAT tissue of CRC patients associated with poor disease prognosis

**Fig. 6:**
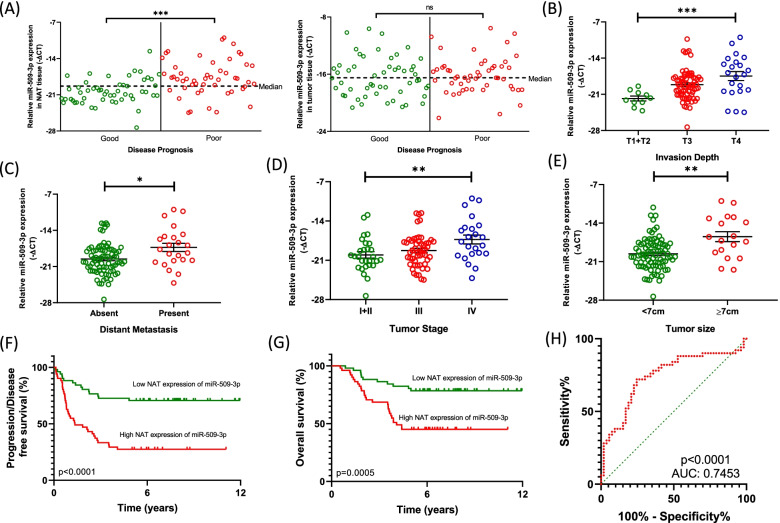
MiR-509-3p is overexpressed in the NAT tissue of patients associated with poor prognosis in CRC. Relative expression of miR-509-3p within the **A** NAT tissue and the tumor tissue in CRC patients exhibiting overall good prognosis versus poor prognosis. The dotted line indicates the median expression of miR-509-3p within the corresponding tissue type. **B**-**E** Correlation of the expression of miR-509-3p within the CRC NAT tissue with the clinicopathological characteristics of CRC patients within the study (*N* = 103) - **B** Invasion depth, **C** Distant metastasis, **D** Tumor stage, and **E** Tumor size. Expression of miR-509-3p was normalized to the expression of RNU6B. Expression results are shown as -∆CT. Kaplan-Meier curves for **F** Disease/progression-free survival, and **G** Overall survival were plotted based on the NAT expression of miR-509-3p in patients with CRC. Expression of miR-509-3p was separated into low and high groups based on the median NAT tissue expression of miR-509-3p. Assessment was performed for a period of 5 years from the date of surgery. **H** Receiver Operating Characteristic (ROC) curve for NAT tissue expression of miR-509-3p was performed to classify CRC patients showing poor prognosis from good prognosis. *p value < 0.05; **p value < 0.01; ***p value < 0.001

Since the overexpression of miR-509-3p within the colorectal tumor tissue was associated with the occurrence of an aggressive disease, we attempted to revisit the profile of the miRNA within the previously used 103 clinical specimens to further explore any variations in the expression pattern of miR-509-3p that may assist in an improved characterization of the miRNA in the process of colorectal carcinogenesis. As a first step, we segregated the patients into two categories – Poor prognosis (*disease recurrence (primary tumor or metastasis)/progression or death within 5 years of surgical excision of the primary tumor*; *N* = 50), and Good prognosis (*disease free overall survival for at least 5 years following primary curative surgery*; *N* = 53). Subsequently, we analyzed the expression of miR-509-3p from the clinical samples (colorectal tumor and NAT tissue) within each patient from both the disease prognosis categories. Interestingly, the expression of miR-509-3p within the NAT tissue was significantly higher in the patients showing poor prognosis as compared to the good prognosis group (*p* < 0.0001) (Fig. 6A). Specifically, 72% (36/50) of the patients from the poor prognosis group showed a miR-509-3p expression that was equal or higher than the global median expression (−∆CT = − 19.329) of the miRNA within the NAT tissues, while only 28.3% (15/53) of patients showing good disease prognosis showed a higher NAT expression of miR-509-3p than its global median. In contrast, the expression of miR-509-3p within the colorectal tumor did not show any statistically significant difference between the Poor versus Good prognosis CRC cases.

We subsequently performed a clinicopathological significance analysis for the NAT expression of miR-509-3p (Supplementary Table 3). Patients presenting with a greater depth of tumor invasion (T4) showed a significantly higher expression of the miRNA within the NAT tissue (median -∆CT = − 17.00), compared to the T3 stage (median -∆CT = − 19.14) and the early stages (T1 & T2) (median -∆CT = − 21.92) (*p* = 0.0009) (Fig. 6B). In addition, 65% (15/23) of the CRC patients with distant metastasis had a NAT miR-509-3p expression greater than the global median, while only 45% (36/80) patients from the non-metastatic group showed a median-high expression of the miRNA within the NAT tissues (*p* = 0.0131) (Fig. 6C). Consequently, a high NAT expression of miR-509-3p was also observed to be associated with an advanced CRC stage presentation (*p* = 0.0075) (Fig. 6D). Lastly, CRC patients presenting with larger primary tumors (≥7 cm) were observed to have a significantly higher NAT tissue expression of miR-509-3p (*p* = 0.0035) (Fig. 6E). None of the other clinical parameters showed any statistically significant correlation with a higher expression of miR-509-3p in the tumor adjacent non-cancerous mucosae. These results indicate that miR-509-3p is overexpressed within the NAT of CRC patients associated with poor disease prognosis.

The prognostic value of miR-509-3p overexpression within the NAT tissue in CRC patients who underwent curative surgery for the primary tumor was determined. Patients with elevated NAT expression of miR-509-3p showed a significantly shorter Disease/Progression-Free Survival (DFS/PFS) (median: 1.35 versus 7.34 years, *p* < 0.0001) (Fig. 6F) and Overall Survival (OS) (median: 4.08 versus 7.50 years, *p* < 0.0001) (Fig. 6G) in comparison with those with a lower miR-509-3p expression. Furthermore, univariate analysis for critical clinicopathological parameters with a potential prognostic significance revealed that the NAT overexpression of miR-509-3p, as well as primary tumor histological grade, invasion depth, distant metastasis, and TNM stage correlated with DFS/PFS and OS (Supplementary Table 4). These five clinical parameters were subsequently applied for multivariate analysis using a Cox proportional hazards regression model, and the results suggested that while distant metastasis was an independent risk factor for DFS/PFS, histological grade and invasion depth were independent risk factors for OS. Remarkably, overexpression of miR-509-3p in the NAT tissue served as an independent risk factor for both, DFS/PFS and OS. Taking this result into account, we also determined the predictive ability of miR-509-3p as a potential prognostic biomarker in CRC using a Receiver Operating Characteristic (ROC) curve. We found that the NAT expression of miR-509-3p in CRC shows a strong statistically significant separation between patients showing poor prognosis versus good prognosis with an Area Under Curve (AUC) of 0.7453 (95% CI: 0.6467–0.8439, *p* < 0.0001) (Fig. 6H).

### Upregulated expression of miR-509-3p within the CRC cell lines has the potential to impact the oncogenic behavior of normal colon cells

**Fig. 7:**
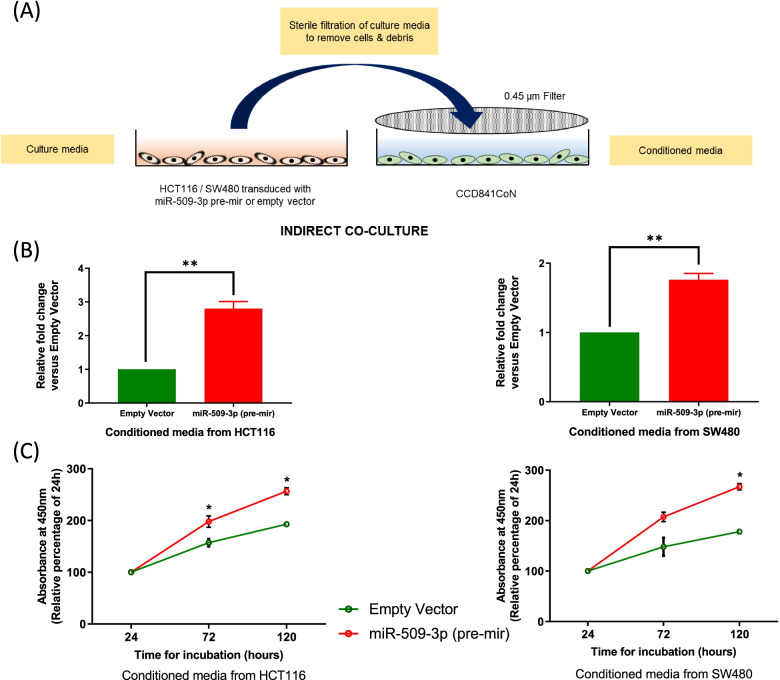
Overexpression of miR-509-3p can be laterally transferred from stably transduced CRC cell lines to a normal colon cell line. **A** Schematic of the experimental transfer of culture media from HCT116 or SW480 CRC cell lines stably transduced with miR-509-3p (pre-mir) or empty vector control through a 0.45 μm filter which serves as a conditioned media for the growth of CCD841CoN, a normal colon cell line. **B** CCD841CoN showed a sharp increase in the expression of miR-509-3p upon being cultured in the conditioned media from HCT116 or SW480 stably transduced with miR-509-3p, as compared to the empty vector control group. **C** Lateral transfer of miR-509-3p from stably transduced HCT116 or SW480 to CCD841CoN resulted in an increase in the proliferation rates of the normal cell line as compared to the empty vector group. Assessment of the proliferation rates were performed using a BrdU assay. *p value < 0.05.**p value < 0.01

The importance of miR-509-3p as a positive regulator of the process of colorectal carcinogenesis was established through clinical observations as well as in vitro and in vivo experiments. While no statistically significant difference between the tumor expression of miR-509-3p in poor prognosis versus good prognosis patients was observed, a high NAT tissue expression of the miRNA correlated with a poor disease prognosis in CRC. Consequently, we aimed to determine if an upregulated expression of miR-509-3p within the CRC cells could influence the oncogenic potential of the adjacent non-tumor cells through in vitro analyses. We opted to implement an indirect co-culture model wherein a 24-h old culture media from HCT116 or SW480 cell lines stably transduced with miR-509-3p (pre-mir), or empty vector control was sterile filtered and was subsequently used to culture a normal colon cell line – CCD841CoN. A schematic of this experimental approach is outlined in Fig. 7A. Sterile filtration of the media ensured that no spillover of cells or debris occurred from the CRC cell lines, while other non-cellular components smaller than 0.45 μm was transferred to the CCD841CoN cell line. Following an exposure period of 24 h to the conditioned media, we profiled the miR-509-3p levels within the CCD841CoN cell line and found a significant overexpression of the miRNA relative to the control. Specifically, conditioned media from HCT116 transduced with miR-509-3p (pre-mir) resulted in a 2.80-fold upregulation of the miRNA (*p =* 0.0045), while media from SW480 overexpressing miR-509-3p caused a 1.76-fold (*p* = 0.0048) increase in the levels of miRNA relative to the negative control (Fig. 7B).

We subsequently attempted to determine if the lateral transfer of miR-509-3p from the CRC cell lines to the normal colon cell line, could also impact its oncogenic potential. Using a BrdU incorporation assay, we studied the proliferative behavior of the CCD841CoN growing under the conditioned media. As is evident from Fig. 7C, exposure of CCD841CoN to conditioned media from CRC cell lines with an upregulated expression of miR-509-3p caused a significant increase in the cell proliferation rates of the normal colon cell line relative to the negative control. While exposure to the conditioned media from HCT116 with elevated miR-509-3p levels increased the proliferative rate of CCD841CoN by 256.63% after 120 h (Control: 192.86% at 120 h, *p =* 0.0019), normal colon cells growing in conditioned media from SW480 with upregulated miR-509-3p showed a 267.04% growth rate at 120 h (Control: 178.14% at 120 h, *p* = 0.0098).

### Lateral transfer of miR-509-3p induces a metastatic phenotype in a normal colon cell line

**Fig. 8:**
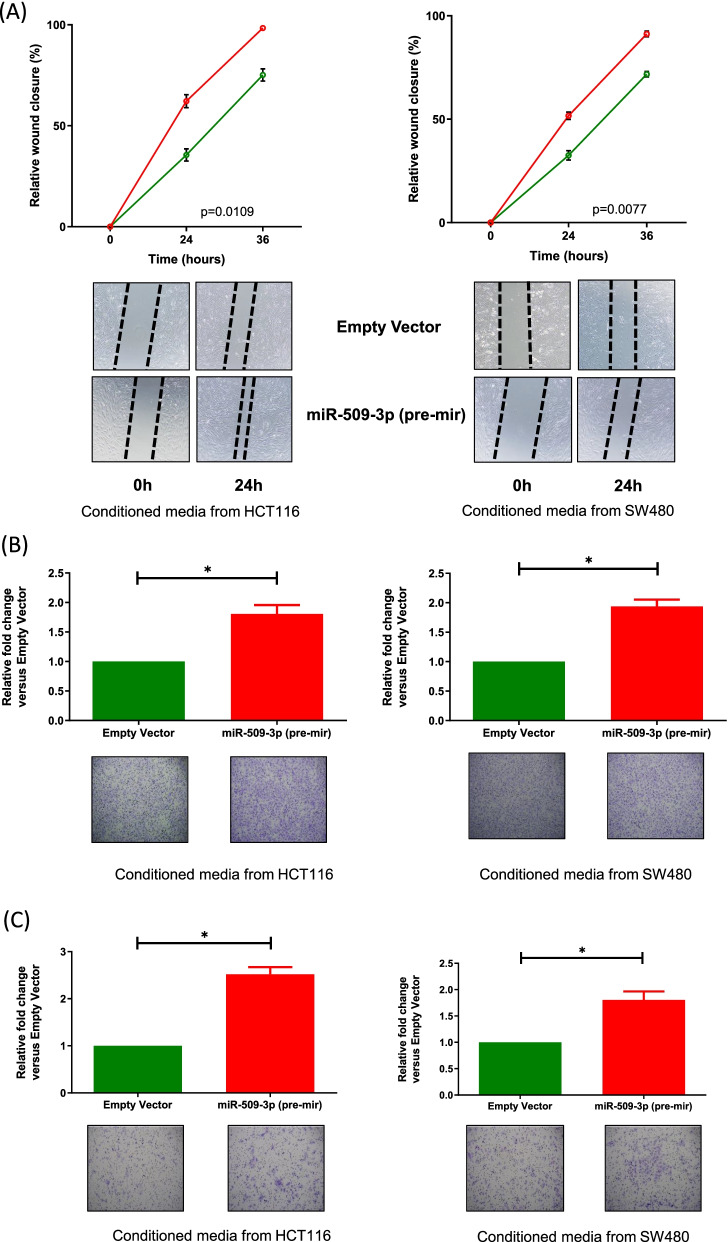
Lateral transfer induced upregulation of miR-509-3p within CCD841CoN induces a metastatic potential within the normal cell line. **A** An increase in the wound closure ability of miR-509-3p overexpressing CCD841CoN was also observed relative to the empty vector group. This increase was seen for CCD841CoN treated with conditioned media from both HCT116 and SW480 cell lines stably transduced with miR-509-3p (pre-mir). **B**-**C** Treatment of CCD841CoN with conditioned media from HCT116 or SW480 stably transduced with miR-509-3p (pre-mir) increased the **B** migration, and **C** invasion rates of CCD841CoN, as compared to the empty vector group. *p value < 0.05

Since miR-509-3p was found to increase the proliferative ability of the normal colon cell line which is one of the critical early steps in the process of carcinogenesis, we wished to assess if the overexpression of the miRNA can channel other aspects of cancer development as well, such as the ability of a cell to metastasize. We used a wound-healing assay to have a preliminary understanding of the migratory ability of the normal colon cell line growing in the conditioned media. Under the influence of conditioned media from HCT116 or SW480 cell line overexpressing miR-509-3p, CCD841CoN showed a significantly higher rate of migration, leading to wound closure (HCT116–98.37% (miR-509-3p) vs. 75.16% (empty vector) wound closure after 36 h; *p =* 0.0109) (SW480–91.20% (miR-509-3p) vs. 71.75% (empty vector) wound closure after 36 h; *p* = 0.0077) (Fig. 8A). We obtained similar results when the conditioned media treated CCD841CoN cells were seeded in a migration chamber and were tested for their ability to migrate towards a chemoattractant. Growing in the presence of sterile-filtered media from HCT116 or SW480 cells stably transduced with miR-509-3p (pre-mir), CCD841CoN cells demonstrated a nearly 2-fold higher rate of migration relative to the negative control (HCT116 – *p* = 0.0335; SW480 – *p* = 0.0148) (Fig. 8B). Moreover, growing in the conditioned media from the CRC cell lines, the normal colon cell line also demonstrated an increased ability of invading through the Matrigel coated migration chamber (Fig. 8C). Specifically, CCD841CoN treated with conditioned media from HCT116 cells overexpressing miR-509-3p demonstrated a 2.51-fold higher rate of invasion relative to the empty vector control (*p* = 0.0101), while colon cells growing in media from SW480 cells stably transduced with miR-509-3p (pre-mir) showed a 1.80-fold increase in the number of invaded cells as compared to the control (*p* = 0.0382).

## Discussion

This study provides the first systematic evidence of novel oncogenic roles of miR-509-3p in CRC. To begin with, we present the earliest report of an overexpression of this miRNA within the colorectal tumor tissues that correlates with an aggressive disease presentation. Our findings specifically indicate that upregulated expression of miR-509-3p is associated with an increase in cell proliferation, migration, and invasion potential in CRC. Importantly, the genomic location of miR-509-3p (pre-miR) is in chromosome X, and X chromosome amplifications have been reported in over 60% of CRC cases [27–29].

It is common knowledge now that the epigenetic regulation of miRNAs occurs through the targeted silencing of mRNA expression in a sequence-specific manner [30]. Indeed, the identification of the functional role of a miRNA as an oncomir or a tumor suppressor is heavily dependent on the nature of its target mRNA. Put simply, a miRNA that is found to inhibit the expression of a tumor suppressor is said to have an oncogenic behavior, while miRNAs suppressing oncogenes are tumor suppressive. In line with this, the identification of the tumor suppressor PHLPP2 as a novel direct target of miR-509-3p within this study strongly favors our preliminary finding of an oncogenic role of the miRNA in CRC. Furthermore, expression analysis of miR-509-3p and PHLPP2 within clinical samples revealed a statistically significant negative correlation between the miRNA and the phosphatase expression in CRC. PHLPP2 belongs to a novel family of Ser/Thr protein phosphatases which functions as a negative regulator of Protein Kinase B (Akt/PKB) and Protein Kinase C (PKC) [31, 32]. Akt/PKB, a major downstream regulator of the phosphoinositide-3-kinase (PI3K) signaling pathway, and PKC are members of the AGC kinase superfamily that upon phosphorylation promote key cell survival signals that ultimately lead to a higher cell proliferation and inhibition of apoptotic pathways [33, 34]. Direct dephosphorylation by *PHLPP2* and the second isoform of PHLPP, *PHLPP1,* incapacitates the pro-oncogenic Akt/PKB and PKC kinases and activates pro-apoptotic genes such as Macrophage-Stimulating 1 (*Mst1)* [31, 35, 36]. Evidence has emerged that the expression of the tumor suppressor PHLPP2 is ubiquitously lost in several cancers, including CRC [37, 38]. Overexpression of PHLPP2 or its isoform is associated with a decreased cell proliferation and tumor development in vitro and in animal models [36, 37, 39]. Dysregulated expression of the phosphatase has also been found to impact the metastatic dissemination of cancer cells by regulating the levels of key members of the EMT pathway, including E-cadherin and Vimentin [39, 40]. The EMT is a biological process that involves the transdifferentiation of a polarized epithelial cell into a mobile mesenchymal cell and is a vital mechanism in cancer progression and metastasis [41, 42]. The process is characterized by a progressive loss of critical epithelial cell morphology and molecular markers such as the tight junction proteins claudin and occludin, adherens junction protein E-cadherin, as well as β-catenin [43]. While a parallel increase is observed in the expression of mesenchymal markers such as Vimentin, neural cadherin (N-Cadherin), fibronectin, α5β1 integrin, and matrix metalloproteinases (MMP) MMP2 and MMP9, that allows the EMT process to continue [41, 43]. Cells undergoing EMT have also been found to be associated with dynamic changes in actin cytoskeleton reorganization including the formation of actin stress fibers under the influence of RhoA, a key member of the Rho Guanosine Triphosphatase (GTPAse) superfamily [44]. Interestingly, overexpression of PHLPP has been shown to be associated with a decrease in the formation of actin stress fibers [40], perhaps through a negative regulation of RhoA via the PI3K-Akt axis [45, 46].

Results from our study confirmed that overexpression of miR-509-3p was associated with an increase in the cell survival and metastatic potential of CRC cells. Moreover, NOD-SCID mice orthotopically injected with miR-509-3p overexpressing CRC cells developed significantly larger primary colonic tumors that also showed a greater metastatic spread to distant organs, relative to the control mice. At the molecular level, upregulation of miR-509-3p was found to potentiate the expression of RhoA with a concurrent decrease in the levels of epithelial marker E-Cadherin – genes critical to the EMT process of cancer progression. Collectively, these results demonstrate the oncogenic behavior of miR-509-3p within CRC which we believe may be largely governed through the direct targeting of PHLPP2 which may subsequently regulate other oncogenic factors within the cell. Exploring the precise mechanisms of this molecular workflow will assist in our improved understanding of the role of miR-509-3p in CRC.

Another critical outcome of this study was the identification of an upregulated expression of the miRNA within the NAT tissue of CRC patients with poor disease prognosis relative to the good prognosis patients. Analogous to the clinical results for miR-509-3p within the tumor tissues, upregulated NAT tissue expression of the miRNA was also found to be associated with a poor disease phenotype. Furthermore, the expression of miR-509-3p within the NAT tissues, but not the colorectal tumor, was found to be an independent predictor of DFS/PFS and OS in CRC patients; thereby highlighting the importance of the miRNA’s NAT expression as a potential prognostic predictor in CRC. Although data from the NAT tissue is commonly used only as a normalizer to determine the differential expression of a given metabolite within the malignant tissue, it is important to question if the concept of a “normal” tissue truly exists within a tumor microenvironment. Not surprisingly, a large-scale analysis of cancer datasets from The Cancer Genome Atlas (TCGA) revealed that the paired normal samples provide more information about disease prognosis than the tumor tissue itself [47]. Several other studies investigating the molecular profile of the NAT tissue and its contribution in the biology of cancer development have reported similar conclusions [48–50]. Multiple theories have been proposed that offer some insight into this behavior of the NAT tissue. The oldest among them is the “field cancerization” theory which suggests that the NAT mucosa exists in a pre-neoplastic state without any detectable phenotypic alterations, alongside the primary tumor, but have a tendency to assist in cancer development, recurrence and/or progression [51, 52]. The “etiologic field effect” theory extends the concept of field cancerization and includes the contribution of several internal and external etiologic factors (dietary, lifestyle, environmental, microbial, hormonal, and genetic factors) that can predispose the tumor microenvironment vulnerable to neoplasia development and progression [53]. On the contrary, research by Sadanandam et al on HER-2 positive breast cancers and adjacent normal tissues concluded that cellular contamination from the primary tumor, and not field cancerization, is the likely cause of cancer adjacent normal tissue’s contribution in local disease recurrence [54]. While it is difficult to ascertain if the overexpression of miR-509-3p within the NAT tissue in our study is a consequence of a single theory, we hypothesized that the primary tumor tissue may play an important role in regulating the molecular architecture of the adjacent normal mucosae. Our hypothesis stems from the observation of positive clinical correlations of miR-509-3p within the NAT with critical primary tumor parameters such as tumor invasion, size, stage, and metastasis. Although field cancerization may be responsible for maintaining the pre-neoplastic state of the NAT tissue, we believe that the aggressiveness of the developing tumor may strongly contribute by disbursing “fuel” to the adjacent normal tissue, in our case a miRNA molecule, that may potentiate disease progression, subsequent recurrences and an overall poor prognosis. Supporting our hypothesis is a study by Aran et al that compared transcriptomic profile of 6506 tissue samples from eight sources including colon, liver, breast, and prostate, comprising of healthy, NAT and cancerous tissues from the TCGA and the Genotype-Tissue Expression (GTEx) project and concluded that the NAT tissue displays a unique molecular profile that lies intermediate to the healthy and the cancerous tissue [55]. Importantly, with experimental observations from patient derived xenograft mouse models the authors conclude that it is not field-cancerization, and rather the signaling from the primary tumor that plays a critical role in shaping the molecular phenotype of the adjacent non-cancerous mucosa. Indeed, through our indirect co-culture experiments we do observe a lateral transfer of the overexpression of miR-509-3p from the CRC cells to a normal colon cell line which subsequently impacts the oncogenic (proliferative and metastatic) behavior of the colon cells. By making use of separate culture vessels for the growth of the CRC cell and the normal colon cells, and sterile filtering the culture media prior to transfer, we ensure zero contamination of the normal colon cells with the cancer cells. Several studies have reported oncogenic transformation of normal non-malignant epithelial cells or fibroblasts by cancerous cell lines in coculture experiments in a wide array of cancers including breast [56], kidney [57], and colon [58]. Furthermore, a multitude of communication mechanisms have been identified by which the tumor cells interact with the normal cells including the use of soluble carrier molecules such as exosomes, microvesicles, apoptotic vesicles, inflammatory molecules, and protein complexes [59], or through mechanical interactions via cell-cell or cell-extracellular matrix linkages [60]. Considering the small size (~ 21 nucleotide) of a miRNA molecule, most of these communication mechanisms are deemed efficient for a successful transfer from the tumor cells to the normal cells. A subsequent investigation into the molecular mechanisms associated with the increase in the levels of miR-509-3p within the NAT tissues, including the communication signals from the primary colorectal tumor, will further our understanding of the importance of NAT in tumor development and progression. Besides, a deeper understanding of the altered molecular behavior of the NAT tissue may also present with potential therapeutic targets for improved disease management.

## Conclusions

This study provides the first systematic characterization of miR-509-3p in CRC. Our findings suggest that the miRNA is overexpressed within the clinical tumor specimens and plays an important role in the proliferation, migration, and invasion of CRC cells. We also provide evidence to show that miR-509-3p directly targets and inhibits the expression of the tumor suppressor PHLPP2. Furthermore, a higher expression of miR-509-3p within the NAT tissues was associated with a progressive disease, recurrence, and early death in CRC patients, highlighting the importance of this miRNA as a potentially predictive biomarker of poor prognosis in CRC. Data from our study also reveals that the aggressive CRC cells may contribute in impacting the oncogenicity of normal colon cells which indicates the existence of an important communication channel between the tumor and its “normal” microenvironment. Taken together, our data suggests that miR-509-3p is an important miRNA within CRC and further investigations within the function and molecular regulation of this miRNA will help us improve our understanding of the biology of CRC development and progression and will also provide an opportunity for the usage of miR-509-3p as a biomarker or a potential therapeutic target in CRC.

## Supplementary Information

**Additional file 1.**

**Additional file 2.**

## Data Availability

All data generated or analyzed during this study are included in this published article [and its supplementary information files].

## Abbreviations

CRC: Colorectal Cancer
PHLPP2: PH domain leucine-rich repeat-containing protein phosphatase 2
3′-UTR: 3′-untranslated regions
NAT: Tumor adjacent normal
RNU6B: U6 small nucleolar RNA
EMT: Epithelial Mesenchymal Transition
E-cadherin: Epithelial cadherin
RhoA: Ras Homolog Family Member A
MORC3: MORC Family CW-Type Zinc Finger 3
ST3GAL3: ST3 Beta-Galactoside Alpha-2,3-Sialyltransferase 3
RRM2B: Ribonucleotide Reductase Regulatory TP53 Inducible Subunit M2B
FN3K: Fructosamine 3 Kinase
TP53INP1: Tumor Protein P53 Inducible Nuclear Protein 1
DEDD: Death Effector Domain Containing Protein
IGF2BP2: Insulin Like Growth Factor 2 MRNA Binding Protein 2
SLC46A1: Solute Carrier Family 46 Member 1
PTEN: Phophatase and Tensin Homolog
CFTR: Cystic Fibrosis Transmembrane Regulator
DFS: Disease free survival
PFS: Progression free survival
