# Supplementary material for: MiR-509-3p is oncogenic, targets the tumor suppressor PHLPP2, and functions as a novel tumor adjacent normal tissue based prognostic biomarker in colorectal cancer
Source: BMC Cancer. 2022 Mar 31;22:351. doi: 10.1186/s12885-021-09075-x (PMC8969217; doi:10.1186/s12885-021-09075-x)
Supplement: Supplementary file 1 — Additional file 1. [file 12885_2021_9075_MOESM1_ESM.pdf]

Supplementary Table 1: Baseline Characteristics of the total CRC study population.

Clinical Parameters	Case distribution (N=103) [N (%)]
**Age (years)**	
<65	52 (50.5)
≥65	51 (49.5)
**Sex**	
Male	63 (61.2)
Female	40 (38.8)
**Tumor Location**	
Colon	70 (68.0)
Rectosigmoid	10 (9.7)
Rectum	23 (22.3)
**Histological grade**	
Well	7 (6.8)
Moderate	90 (87.4)
Poor	6 (5.8)
**Invasion depth**	
T1+T2	10 (9.7)
T3	71 (68.9)
T4	22 (21.4)
**Lymph Node metastasis**	
Absent	34 (33.0)
Present	69 (67.0)
**Distant Metastasis**	
Absent	80 (77.7)
Present	23 (22.3)
**TNM stage**	
I+II	28 (27.2)
III	52 (50.5)
IV	23 (22.3)
**Tumor size**	
<7cm	85 (82.5)
≥7cm	18 (17.5)

Supplementary Table 2: Correlation of the expression of miR-509-3p in the tumor tissue within the CRC study population and the patient clinicopathological characteristics.

Clinical Parameters	Case distribution (N=103) [N (%)]	Relative miR-509-3p expression in tumor tissue (-∆CT) [Median (Range)]	p-value
**Age (years)**			
<65	52 (50.5)	-16.50 (-20.68 - -9.56)	0.5477
≥65	51 (49.5)	-16.58 (-22.13 - -10.32)	
**Sex**			
Male	63 (61.2)	-16.23 (-20.36 - -9.56)	0.9026
Female	40 (38.8)	-16.60 (-22.13 - -9.63)	
**Tumor Location**			
Colon	70 (68.0)	-16.43 (-22.13 - -9.63)	0.5132
Rectosigmoid	10 (9.7)	-16.41 (-20.13 - -9.56)	
Rectum	23 (22.3)	-17.24 (-20.68 - -10.18)	
**Histological grade**			
Well	7 (6.8)	-17.24 (-20.24 - -14.96)	0.4758
Moderate	90 (87.4)	-16.50 (-22.13 - -9.56)	
Poor	6 (5.8)	-16.54 (-18.11 - -14.27)	
**Invasion depth**			
T1+T2	10 (9.7)	-18.52 (-22.13 - -12.24)	0.0356*
T3	71 (68.9)	-16.83 (-20.68 - -9.56)	
T4	22 (21.4)	-15.11 (-21.59 - -9.63)	
**Lymph Node metastasis**			
Absent	34 (33.0)	-17.51(-22.13 - -10.18)	0.0251*
Present	69 (67.0)	-16.18(-21.59 - -9.56)	
**Distant Metastasis**			
Absent	80 (77.7)	-16.85 (-22.13 - -10.32)	0.0030*
Present	23 (22.3)	-14.93 (-20.36 - -9.56)	
**TNM stage**			
I+II	28 (27.2)	-18.18 (-22.13 - -10.32)	0.0003*
III	52 (50.5)	-16.51 (-21.59 - -10.77)	
IV	23 (22.3)	-14.93 (-20.36 - -9.56)	
**Tumor size**			
<7cm	85 (82.5)	-16.83 (-22.13 - -9.63)	0.0138*
≥7cm	18 (17.5)	-15.27 (-21.59 - -9.56)	

Supplementary Table 3: Correlation of the expression of miR-509-3p in the tumor adjacent normal tissue within the CRC study population and the patient clinicopathological characteristics.

Clinical Parameters	Case distribution (N=103) [N (%)]	Relative miR-509-3p expression in tumor tissue (-ΔCT) [Median (Range)]	p-value
**Age (years)**			
<65	52 (50.5)	-19.67(-24.36 - -9.98)	0.8259
≥65	51 (49.5)	-19.06 (-27.35 - -12.60)	
**Sex**			
Male	63 (61.2)	-19.06 (-24.29 - -10.31)	0.2031
Female	40 (38.8)	-20.17 (-27.35 - -9.98)	
**Tumor Location**			
Colon	70 (68.0)	-19.29 (-27.35 - -9.98)	0.6976
Rectosigmoid	10 (9.7)	-19.65 (-22.55 - -10.31)	
Rectum	23 (22.3)	-19.43 (-23.66 - -16.38)	
**Histological grade**			
Well	7 (6.8)	-20.92 (-23.49 - -16.38)	0.1187
Moderate	90 (87.4)	-19.62 (-27.35 - -10.31)	
Poor	6 (5.8)	-17.46 (-19.43 - -9.98)	
**Invasion depth**			
T1+T2	10 (9.7)	-21.92 (-24.17 - -19.43)	0.0009*
T3	71 (68.9)	-19.14 (-27.35 - -10.31)	
T4	22 (21.4)	-17.00 (-24.47 - -9.98)	
**Lymph Node metastasis**			
Absent	34 (33.0)	-20.33 (-27.35 - -12.92)	0.2196
Present	69 (67.0)	-19.06 (-24.47 - -9.98)	
**Distant Metastasis**			
Absent	80 (77.7)	-19.79 (-27.35 - -12.60)	0.0131*
Present	23 (22.3)	-17.69 (-24.17 - -9.98)	
**TNM stage**			
I+II	28 (27.2)	-20.39 (-27.35 - -12.92)	0.0075*
III	52 (50.5)	-19.34 (-24.47 - -12.60)	
IV	23 (22.3)	-17.69 (-24.17 - -9.98)	
**Tumor size**			
<7cm	85 (82.5)	-19.81 (-27.35 - -11.16)	0.0035*
≥7cm	18 (17.5)	-16.85 (-22.48 - -9.98)	

Supplementary Table 4: Univariate and Multivariate analysis of clinicopathological and prognostic significance of miR-509-3p in CRC.					
**Variable**	**Subset**	***Disease/Progression free survival***		***Overall survival***	
		**Hazard ratio (95% CI)**	***P* value**	**Hazard ratio (95% CI)**	***P* value**
Univariate analysis (N=102)					
Age	≥65 years <65 versus years	0.7982 (0.4633 – 1.3750)	0.4162	0.8553 (0.4566 – 1.6020)	0.6258
Sex	Female versus Male	0.9255 (0.5318 – 1.6110)	0.7855	1.1180 (0.5863 – 2.1320)	0.7313
Tumor Location	Colon versus Rectum, Rectosigmoid	1.5510 (0.8744 – 2.7530)	0.1655	1.7880 (0.9259 – 3.4550)	0.1203
Histological grade	Poor versus Moderate, Well	3.4450 (0.8083 – 14.6800)	0.0022*	4.2920 (0.7431 – 24.7900)	0.0009*
Invasion depth	T4 versus T1, T2, T3	2.9410 (1.3810 – 6.2640)	<0.0001*	2.7370 (1.1870 – 6.3140)	0.0016*
Lymph node metastasis	Present versus Absent	1.5260 (0.8662 – 2.6890)	0.1716	1.4830 (0.7737 – 2.8430)	0.2641
Distant metastasis	Present versus Absent	3.4660 (1.5930 – 7.5440)	<0.0001*	2.8990 (1.2750 – 6.5940)	0.0007*
TNM Stage	III, IV versus I, II	2.3210 (1.3000 – 4.1430)	0.0174*	2.5830 (1.3180 – 5.0630)	0.0261*
Tumor size	≥7cm versus <7cm	1.5500 (0.7204 – 3.3370)	0.1920	1.9570 (0.8123 – 4.7170)	0.0616
miR-509-3p (NAT)	High expression versus Low expression	3.5260 (2.0290 – 6.1250)	<0.0001*	3.1890 (1.6950 – 5.9970)	0.0005*
miR-509-3p (tumor)	High expression versus Low expression	1.1270 (0.6539 – 1.9430)	0.6651	1.0060 (0.5366 – 1.8840)	0.9863
Multivariate analysis (N=102)					
Histological grade	Poor versus Moderate, Well	2.3282 (0.9416 – 5.7563)	0.0672	3.2452 (1.1461 – 9.1890)	0.0266*
Invasion depth	T4 versus T1, T2, T3	1.7566 (0.8418 – 3.6654)	0.1333	2.3661 (1.0671 – 5.2464)	0.0340*
Distant metastasis	Present versus Absent	2.3825 (1.1107 – 5.1103)	0.025*	1.3798 (0.6232 – 3.0548)	0.4271
TNM Stage	III, IV versus I, II	1.3640 (0.6210 – 2.9973)	0.4390	1.6959 (0.6611 – 4.3500)	0.2717
miR-509-3p (NAT)	High expression versus Low expression	3.0020 (1.5884 – 5.6737)	0.0007*	2.2903 (1.0946 – 4.7919)	0.0277*

N=102 (Survival data was unavailable for 1 patient)

Supplementary Table 5: Primer sequences used for reverse transcription (RT) and quantitative polymerase chain reaction (QPCR)		
**Gene name**	**Direction**	**Primer sequence (5'-3')**
Oligo dT for reverse transcription	-	CAGGTCCAGTTTTTTTTTTTTTTTVN
hsa-miR-509-3p	Forward	CGCAGTGATTGGTACGTCTG
	Reverse	CCAGTTTTTTTTTTTTTTTCTACCCA
RNU6B	Forward	CGCTTCGGCAGCACATATACTA
	Reverse	ACGCTTCACGAATTTGCGT
MORC3	Forward	TCTTCTCCTAAGGAAAGTGTTCCA
	Reverse	GAGGAACGAGTAGAAAGTCTCCG
ST3GAL3	Forward	GCCTGCTGAATTAGCCACCAAG
	Reverse	CCACTTGCGAAAGGAGTCATCC
RRM2B	Forward	TGCTGTCGTAGTTGGAGGTG
	Reverse	ATGATCTCTCATCCTGATCCAGC
FN3K	Forward	GGCTACAGGTGAAGATCCCG
	Reverse	TAGAAGGAAGCCGGGTCGTA
TP53INP1	Forward	AAGACTCACGGGCACAGAAGTG
	Reverse	TCTTTTATCCACTGGGAAGGGC
DEDD	Forward	AAGTTTCTTGCGGAGTACGGT
	Reverse	TGCTCACCATGCTCTTCTGG
IGF2BP2	Forward	AGTGGGAGGTGTTGGATGGA
	Reverse	GTTGACAACGGCGGTTTCTG
SLC46A1	Forward	TGTTCACAGGATATGGGTTGCTT
	Reverse	ACAGGCCACAGCAGAAAAGA
PTEN	Forward	TGTAAAGCTGGAAAGGGACGA
	Reverse	GGAATAGTTACTCCCTTTTTGTCTC
PHLPP2	Forward	TGGTAGAGCACATCCCCCTC
	Reverse	AGTGCAGGCGGATGGTAAAG
GAPDH	Forward	AATCCCATCACCATCTTCCA
	Reverse	TGGACTCCACGACGTACTCA
