# Supplementary material for: MiR-509-3p is oncogenic, targets the tumor suppressor PHLPP2, and functions as a novel tumor adjacent normal tissue based prognostic biomarker in colorectal cancer
Source: BMC Cancer. 2022 Mar 31;22:351. doi: 10.1186/s12885-021-09075-x (PMC8969217; doi:10.1186/s12885-021-09075-x)
Supplement: Supplementary file 2 — Additional file 2. [file 12885_2021_9075_MOESM2_ESM.pdf]

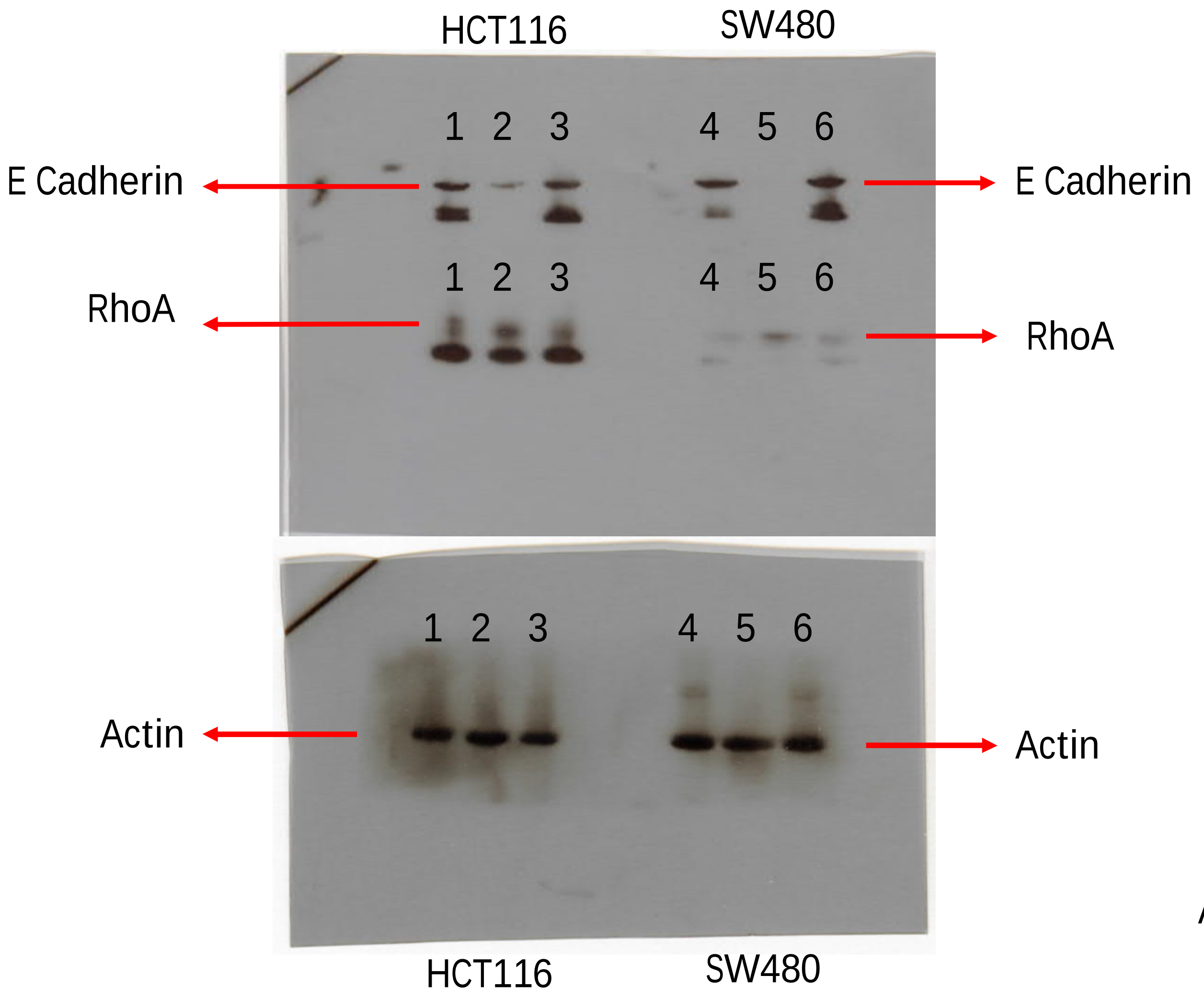

1. Empty Vector
2. miR-509-3p (pre-mir)
3. miR-509-3p (pre-mir) + Anti miR-509-3p
4. Empty Vector
5. miR-509-3p (pre-mir)
6. miR-509-3p (pre-mir) + Anti miR-509-3p

Arrows indicate the target size bands
